# Generation of novel patient-derived ***CIC-****DUX4* sarcoma xenografts and cell lines

**DOI:** 10.1038/s41598-017-04967-0

**Published:** 2017-07-05

**Authors:** Rieko Oyama, Mami Takahashi, Akihiko Yoshida, Marimu Sakumoto, Yoko Takai, Fusako Kito, Kumiko Shiozawa, Zhiwei Qiao, Yasuhito Arai, Tatsuhiro Shibata, Yoshihiro Araki, Makoto Endo, Akira Kawai, Tadashi Kondo

**Affiliations:** 1 Department of Innovative Seeds Evaluation, National Cancer Center Research Institute, 5-1-1 Tsukiji, Chuo-ku Tokyo, 104-0045 Japan; 2 Central Animal Division, National Cancer Center Research Institute, 5-1-1 Tsukiji, Chuo-ku Tokyo, 104-0045 Japan; 3 Department of Pathology and Clinical Laboratories, National Cancer Center Hospital, 5-1-1 Tsukiji, Chuo-ku Tokyo, 104-0045 Japan; 4 Division of Rare Cancer Research, National Cancer Center Research Institute, 5-1-1 Tsukiji, Chuo-ku Tokyo, 104-0045 Japan; 5 Division of Cancer Genomics, National Cancer Center Research Institute, 5-1-1 Tsukiji, Chuo-ku Tokyo, 104-0045 Japan; 6 Division of Musculoskeletal Oncology, National Cancer Center Hospital, 5-1-1 Tsukiji, Chuo-ku Tokyo, 104-0045 Japan

## Abstract

*CIC-DUX4* sarcoma (CDS) is a group of rare, mesenchymal, small round cell tumours that harbour the unique *CIC-DUX4* translocation, which causes aberrant gene expression. CDS exhibits an aggressive course and poor clinical outcome, thus novel therapeutic approaches are needed for CDS treatment. Although patient-derived cancer models are an essential modality to develop novel therapies, none currently exist for CDS. Thus, the present study successfully established CDS patient-derived xenografts and subsequently generated two CDS cell lines from the grafted tumours. Notably, xenografts were histologically similar to the original patient tumour, and the expression of typical biomarkers was confirmed in the xenografts and cell lines. Moreover, the xenograft tumours and cell lines displayed high Src kinase activities, as assessed by peptide-based tyrosine kinase array. Upon screening 119 FDA-approved anti-cancer drugs, we found that only actinomycine D and doxorubicin were effectively suppress the proliferation among the drugs for standard therapy for Ewing sarcoma. However, we identified molecular targeting reagents, such as bortezomib and crizotinib that markedly suppressed the growth of CDS cells. Our models will be useful modalities to develop novel therapeutic strategies against CDS.

## Introduction

*CIC-DUX4* sarcoma (CDS) is a recently characterized subset of high-grade sarcoma that accounts for the majority of Ewing-like small round cell sarcomas^1^. While Ewing sarcomas harbour specific gene fusions that connect *EWSR1* to one of the ETS gene family members^2, 3^, CDS is defined by a gene fusion between the *CIC* and *DUX4* genes^4–8^. Current therapeutic strategy for small round cell sarcoma consists of chemotherapy, radiotherapy, and surgical resection^9–11^; however, a standard therapy for CDS has yet to be established. CDS exhibits a more aggressive clinical course than Ewing sarcoma and quickly acquires chemoresistance^8^, resulting in shorter overall patient survival^12^. Thus, novel therapeutic strategies are required for CDS treatment.

The functions of *CIC–DUX4* fusion gene have been investigated *in vitro*, to elucidate the biological basis of CDS carcinogenesis and development. *CIC* encodes a transcriptional repressor with a high-mobility group (HMG)-box containing DNA-binding domain that functions as a primary downstream sensor of receptor tyrosine kinase (RTK)/extracellular signal-regulated kinase (ERK) pathway activity^13–17^. *DUX4* encodes a double homeodomain transcriptional activator of paired-like homeodomain transcription factor 1 (PITX1)^18–20^. The deduced chimeric *CIC–DUX4* protein exhibits strong transcriptional activity to induce a unique gene expression profile^4^, which considerably differs from that of Ewing sarcoma^21^. Beside these analyses, no study has characterised the detailed biological features of CDS tumours or the possibility of targeting the *CIC–DUX4* fusion protein for CDS therapy.

Patient-derived cancer models are an essential modality to understand molecular carcinogenesis and develop novel therapeutic strategies^22^. However, patient-derived cancer models have not been generated for CDS as of yet. Here, we developed patient-derived cancer models of CDS, characterised their histological and biomolecular features, and investigated the growth inhibitory effects of anti-cancer drugs on CDS cells. To our best knowledge, this is the first report on the establishment of patient-derived cancer models of CDS, and has the potential to facilitate CDS research.

## Results

### Clinical background and tumour histology

**Figure 1:**
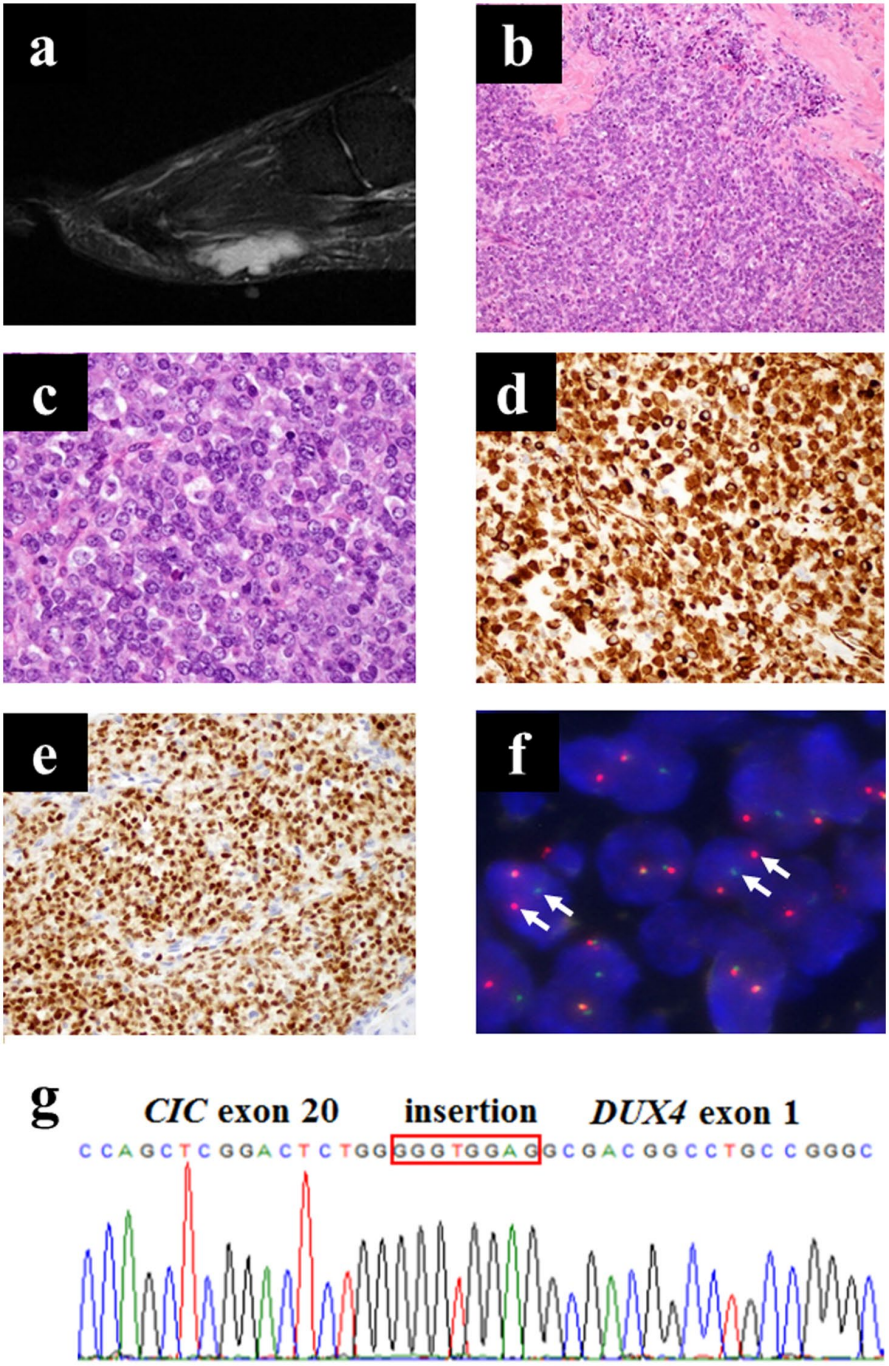
Appearance of the primary tumour. (**a**) T2-weighted MRI image. (**b,c**) H&E stained sections of primary tumour tissues (b, ×200; c, ×600). (**d,e**) Immunohistochemical analysis of WT1 (d) and ETV4 (e). (**f**) *CIC* break-apart FISH of CDS cells (f, arrows indicate the splits). (**g**) Sanger sequence analysis of the transcript showing *CIC–DUX4* fusion. An antiparallel 4767–4774 region of *CIC* exon insertion is indicated in the orange box.

Tumour tissue was obtained from a 29-year-old female diagnosed with CDS and showed multinodular appearance in the superficial layer of soft-tissue. T2-weighted short tau inversion recovery (STIR) sequences of magnetic resonance imaging (MRI) showed a multinodular soft tissue mass in the sole of the foot (Fig. 1a). Tumour tissue consisted of lobulated sheets of small round cancer cells within a sclerotic stroma. The malignant cells were relatively uniform in shape with slightly pleomorphic nuclei and focal prominent nucleoli. (Fig. 1b and c). Immunohistochemical analysis revealed Wilms tumour 1 (WT1) expression throughout the cytoplasm and nucleus (Fig. 1d) and diffuse nuclear ETV4 staining (Fig. 1e). Fluorescence *in situ* hybridization (FISH) experiments indicated that most of the tumour cells harboured split green and orange signals, suggesting the presence of *CIC* gene rearrangement (Fig. 1f), which was then confirmed by RT-PCR. Sanger sequencing identified an in-frame *CIC–DUX4* fusion transcript in which parts of *CIC* exon 20 and *DUX4* exon 1 were fused with the junction nucleotide sequence 5’-GGGTGGAG-3’ (Fig. 1g). Another *CIC–DUX4* fusion transcript connecting a part of *CIC* exon 20 to the *DUX4* 3’-UTR was also detected. Moreover, an Ala-Ser duplication was identified in *DUX4* of the in-frame fusion transcript, which was found only in *DUX4* (4q)—and not in *DUX4L* (10q)—in the hg38 human reference database, suggesting that *CIC* was fused to *DUX4* (4q) (see Supplementary Fig. S1). Collectively, these data suggest that the cells displayed the typical genetic features of CDS.

### Growth of tumour xenografts

**Figure 2:**
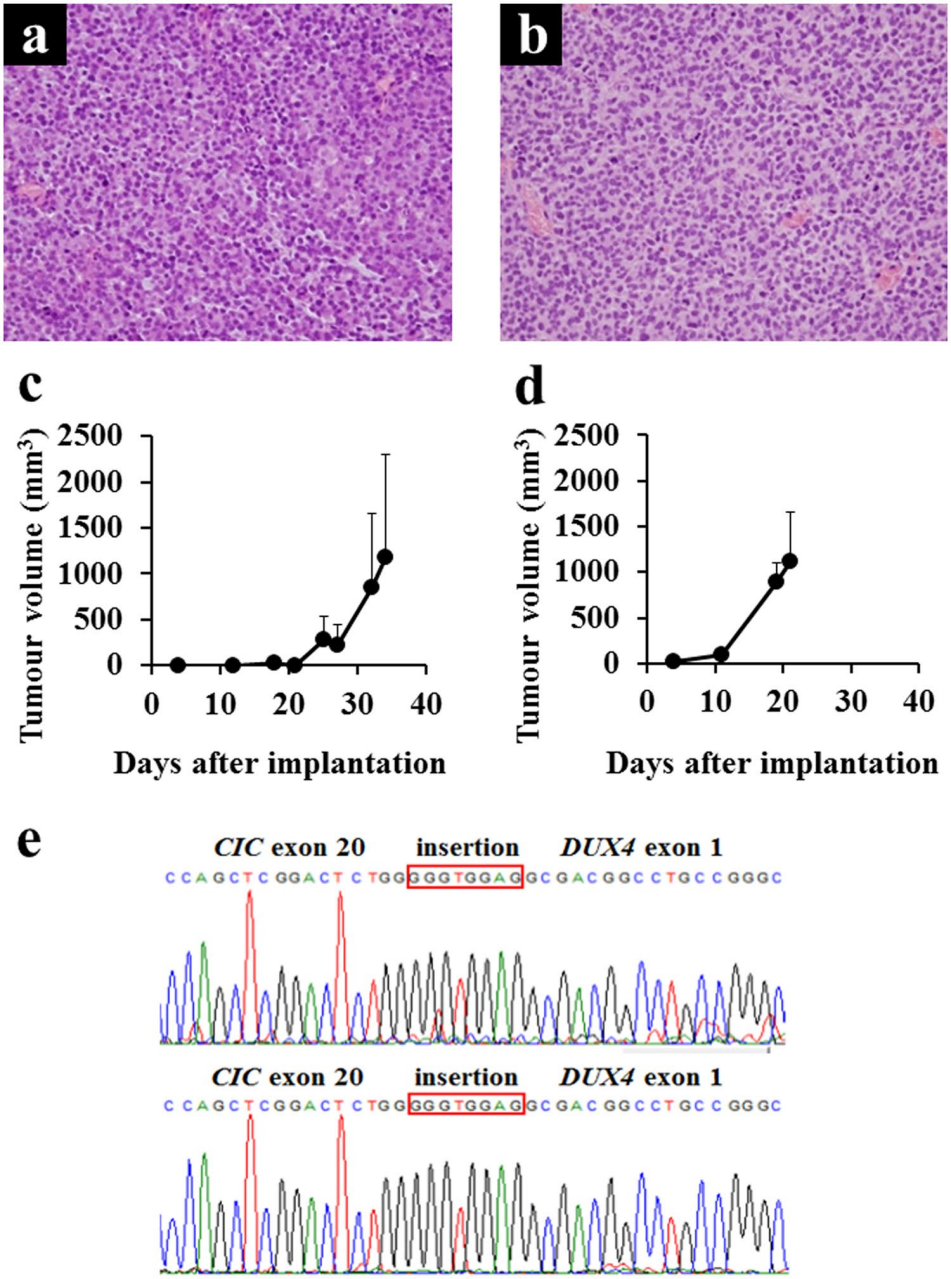
Characteristics of xenograft tumours. (**a,b**) Histology of xenograft tumours (a, NCC-CDS1-X1; b, NCC-CDS1-X3, ×400). (**c,d**) The growth curves of xenograft tumours (c, NCC-CDS1-X1; d, NCC-CDS1-X3). Data represent mean ± SD. (**e**) Sanger sequence analysis of the transcript showing *CIC–DUX4* fusion (upper panel, NCC-CDS1-X1; lower, NCC-CDS1-X3). An antiparallel 4767–4774 region of *CIC* exon insertion is indicated in the orange box.

The patient-derived tumour tissue was subcutaneously grafted into immunodeficient mice, propagated *in vivo*, and then serially transplanted three times. Tumour histology at the first and third transplantation passages is shown in Fig. 2a and b, respectively. The morphological features were quite similar between the two passages, and to the primary tumour tissue. In the first-passage xenografts, a tumour started to increase in size 30 days after transplantation (Fig. 2c). In the third passage, the tumour grew 2 weeks after transplantation (Fig. 2d). Third-passage xenograft tissues were frozen and later inoculated in mice, which confirmed successful tumour propagation after prolonged storage. RT-PCR and Sanger sequencing confirmed presence of the same *CIC-DUX4* transcript as in the primary tumour (Fig. 2e).

### Characterisation of the CDS cell lines

**Figure 3:**
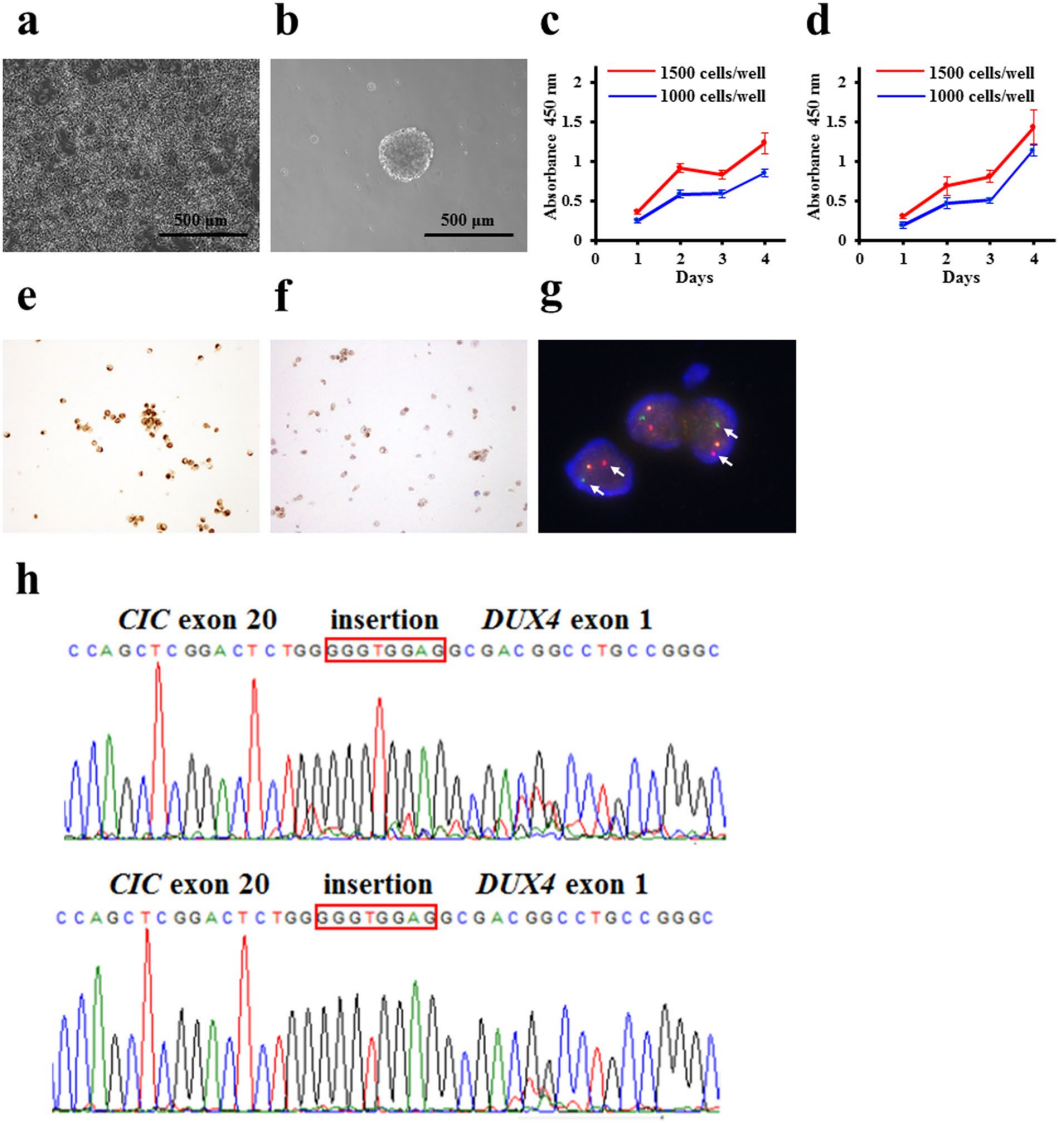
Characteristics of CDS cells. (**a**) Phase-contrast images of CDS cells cultured in conventional culture conditions and (**b**) in the ultra-low attachment culture dishes. Bar: 500 μm. (**c,d**) The growth curves for NCC-CDS1-X1-C1 (c) and X3-C1 (d) cells. (**e,f**) Immunohistochemical analysis for WT1 (e) and ETV4 (f). (**g**) *CIC* break-apart FISH of CDS cells. The arrows indicate the splits. (**h**) Sanger sequence analysis of the transcript showing *CIC–DUX4* fusion (upper, NCC-CDS1-X1-C1; lower, NCC-CDS1-X3-C1). An antiparallel 4767–4774 region of *CIC* exon insertion is indicated in the orange box.

Tumour cells were recovered from the first and third xenograft passages and seeded onto culture dishes. Small round cells were observed a few days after seeding, and were cultured for more than 50 passages (more than 12 months, Fig. 3a). Five days after seeding on a low attachment surface dish, cells formed well-round spheroids (Fig. 3b). Population doubling time during logarithmic growth phase was approximately 40 h and 30 h for cells established from the first and third passages, respectively (Fig. 3c and d). The appearance and growth characteristics of the two cell lines were quite similar, and no considerable differences were observed between the two cell lines.

The histological biomarkers for CDS were expressed consistently in the two cell lines. Specifically, immunohistochemical staining revealed the expression of WT1 and ETV4 in nuclei (Fig. 3e and f, respectively), as well as CD99, and vimentin (see Supplementary Fig. S2). We did not observe the expression of myogenin, or S100 antigen (Supplementary Fig. S2). Short tandem repeat (STR) patterns of alleles were identical in the primary tumour, xenograft tumours, and cultured cell lines (Supplementary Table S1), and distinct from any other cell lines deposited in public cell banks—such as the Japanese Collection of Research Bioresources Cell Bank (JCRB, http://jcrbcelldata.nibiohn.go.jp) and Deutsche Sammlung von Mikroorganismen und Zellkulturen (DSMZ, http://www.dsmz.de).

### Detection of *CIC–DUX4* fusion transcripts in cell lines

The presence of *CIC* rearrangement was confirmed in the NCC-CDS1-X1-C1 cells by FISH. A split green and orange fused signal was consistent with balanced translocation (Fig. 3g). RT-PCR and Sanger sequencing confirmed presence of the *CIC-DUX4* in-frame gene fusion in two cell lines ﻿as well as in﻿ the primary tumour and two xenograft tumours (Fig. 3h).

### Phosphorylation analysis in CDS cell lines

**Figure 4:**
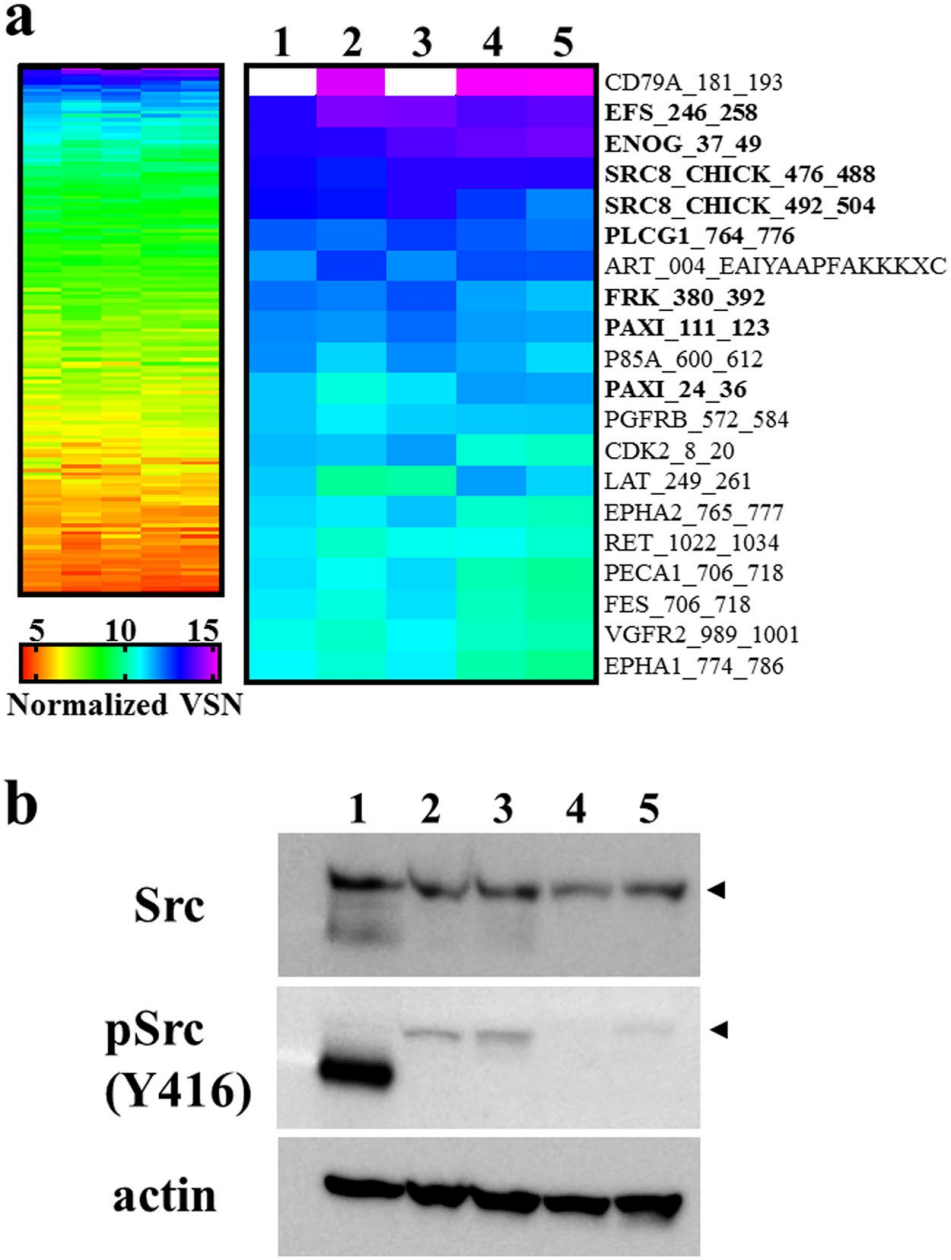
Tyrosine kinase activity in primary tumour, xenograft tumours, and the cell lines. (**a**) Tyrosine kinase activity detected with an array of peptide substrate is fully shown in the left panel and the signal intensities of the top 20 peptides are shown in the right panel. 1: tumour tissue, 2: NCC-CDS1-X1 tumour tissue, 3: NCC-CDS1-X3 tumour tissue, 4: NCC-CDS1-X1-C1 cells, 5: NCC-CDS1-X3-C1 cells. (**b**) Western blotting analysis of Src. Arrowheads indicate the position of Src with a molecular weight of 60 kDa. Full-length blots are shown in Supplementary Fig. S3.

A previous study demonstrated that CIC functions as a repressor of RTK-responsive genes in the absence of signalling. When the RTK signalling is activated, CIC silencing is relieved, and the targeted gene is expressed in response to local or ubiquitous activators^23^. On the basis of this information, we screened for kinase activity, using cell lysates and peptide-based tyrosine kinase arrays^24^. The overall patterns of kinase activity were mostly similar among the primary tumour, xenograft tumours, and cell lines (Fig. 4a, left). Top 20 highest signal peptides that were consistently phosphorylated by the kinases in the different groups are shown in the right panel of Fig. 4a. The sequences are listed in Table 1. Notably, five peptides mimetics of non-receptor tyrosine kinases of the Src family substrates were prominently and consistently phosphorylated in all samples examined as follows: EFS_246_258 (GGTDEGIYDVPLL), SRC8_CHICK_492_504 (YQAEENTYDEYEN), SRC8_CHICK_476_488 (EYEPETVYEVAGA), PLCG1_764_776 (IGTAEPDYGALYE), FRK_380_392 (KVDNEDIYESRHE), PAXI_111_123 (VGEEEHVYSFPNK), and PAXI_24_36 (FLSEETPYSYPTG) (indicated by bold characters in Fig. 4a). To confirm Src kinase activity in the lysates, we examined Src (Y416) phosphorylation status by western blotting (Fig. 4b, full-length blots are presented in Supplementary Fig. S3). Src was consistently expressed in the primary tumour tissue, the two xenograft tumours, and cell lines, although the expression was slightly reduced in the latter. Moreover, Src (Y416) was also phosphorylated in the primary tumour, two xenograft tumours, and one of the two cell lines. Thus, while the kinase activities were similar among the primary tumour, xenograft tumours, and cell lines, there may be slight differences in expression or activity among these entities.

**Table 1:** Tyrosine kinase substrates on the peptide array and the corresponding kinases.

Array ID	Sequence	UniProt ID	Position	Kinase
**CD79A_181_193**	EYEDENLYEGLNL	P11912	Y188	SRC-type Tyr-kinases Lyn
**EFS_246_258**	GGTDEGIYDVPLL	O43281	Y253	Src
**ENOG_37_49**	SGASTGIYEALEL	P09104	Y44	—
**SRC8_CHICK_476_488**	EYEPETVYEVAGA	Q01406	Y483	Src, PTK2
**SRC8_CHICK_492_504**	YQAEENTYDEYEN	Q01406	Y499	Src
PLCG1_764_776	IGTAEPDYGALYE	P19174	Y775	EGFR, Syk
FRK_380_392	KVDNEDIYESRHE	P42685	Y387	Tyrosine-protein kinase FRK
PAXI_111_123	VGEEEHVYSFPNK	P49023	Y118	PTK6, PTK2
**P85A_600_612**	NENTEDQYSLVED	P27986	Y607	Src, Insr, PI3K
PAXI_24_36	FLSEETPYSYPTG	P49023	Y31	PTK6, PTK2
PGFRB_572_584	VSSDGHEYIYVDP	P09619	Y581	PDGFRB
CDK2_8_20	EKIGEGTYGVVYK	P24941	Y15	Wee1-like protein kinase
LAT_249_261	EEGAPDYENLQEL	O43561	Y255	—
EPHA2_765_777	EDDPEATYTTSGG	P29317	Y772	—
RET_1022_1034	TPSDSLIYDDGLS	P07949	Y1029	Proto-oncogene tyrosine-protein kinase receptor Ret
PECA1_706_718	KKDTETVYSEVRK	P16284	Y713	Tyrosine-protein kinase Fer
FES_706_718	REEADGVYAASGG	P07332	Y713	Tyrosine-protein kinase Fes/Fps
VGFR2_989_1001	EEAPEDLYKDFLT	P35968	Y996	VEGFR-2
EPHA1_774_786	LDDFDGTYETQGG	P21709	Y781	Ephrin type-A receptor 1

Tyrosine kinases were predicted by screening Phospho.elm (http://phospho.elm.eu.org) and Uniprot (http://www.uniprot.org) databases.

### Proteomic annotation study

**Figure 5:**
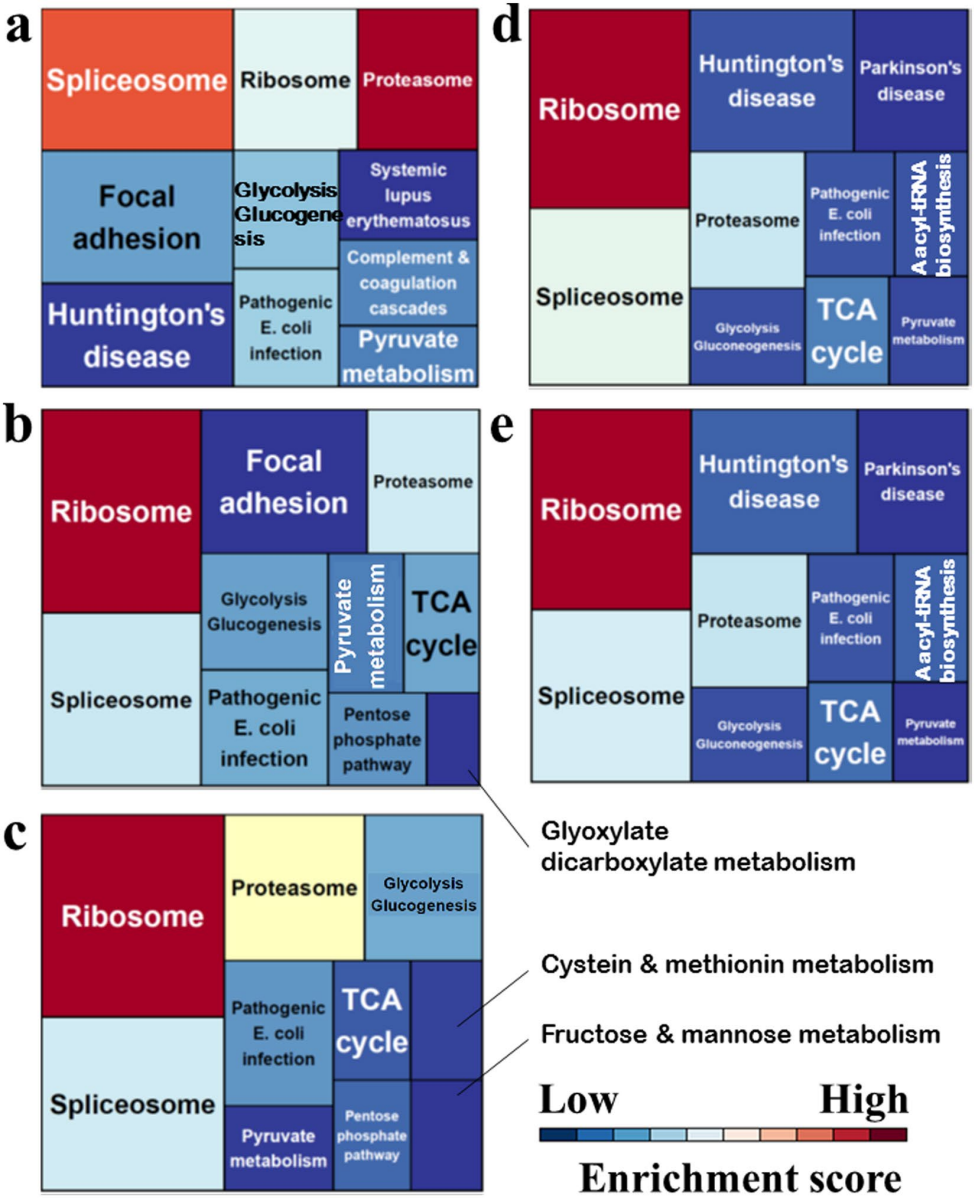
Treemaps of KEGG pathway categories for enrichment analyses of (**a**) primary tumour tissue, (**b**) NCC-CDS-X1, (**c**) NCC-CDX-X3, (**d**) NCC-CDS1-X1-C1, and (**e**) NCC-CDS1-X3-C1. The box sizes represent the numbers of proteins in that category, and colours represent the enrichment scores. All process groups were considered significant at p < 0.05.

Protein expression profiles of the primary tumour, two xenograft tumours, and two cell lines were obtained by mass spectrometry (see Supplementary Dataset 1–5). The proteins identified in the individual samples were classified according to their possible functions based on the data from the Kyoto Encyclopedia of Genes and Genomes (KEGG) database, and the top 10 most enriched pathways are presented in a treemap format (Fig. 5), where area size is parallel to the number of proteins allocated, and the colours indicate the degree of statistical enrichment. In the primary tumour, proteins in the spliceosome pathway were most dominantly detected and the proteasome pathway was the most enriched (Fig. 5a). In the xenograft tumours, the overall appearance of the treemap was quite different from that of the primary tumour. Proteins from the ribosome pathway were mostly detected, and the ribosome pathway was the most significantly enriched pathway (Fig. 5b,c). The enrichment score for the proteasome and spliceosome was decreased in the xenograft tumours compared to those in the original tumour, and the enrichment score of the spliceosome decreased in the two xenograft tumours (Fig. 5b,c). Additionally, the focal adhesion pathway proteins were the third most detected in the primary tumour, although the degree of enrichment decreased in the first passage xenograft, and disappeared from the top 10 most enriched pathways of the third passage xenograft (Fig. 5a–c). In the two cell lines, the ribosome, spliceosome, and proteasome pathways were commonly enriched, as well as in the two xenograft tumours (Fig. 5b–e). The focal adhesion pathway did not appear in the cell lines (Fig. 5d,e). Thus, these results suggest that protein expression profiles were transiently changed during the course of cancer model establishment. The spectrum of pathways in primary and xenograft tumour tissue and the established cell lines are summarized in Supplementary Table S2.

### Screening of anticancer drugs in CDS cell lines

**Figure 6:**
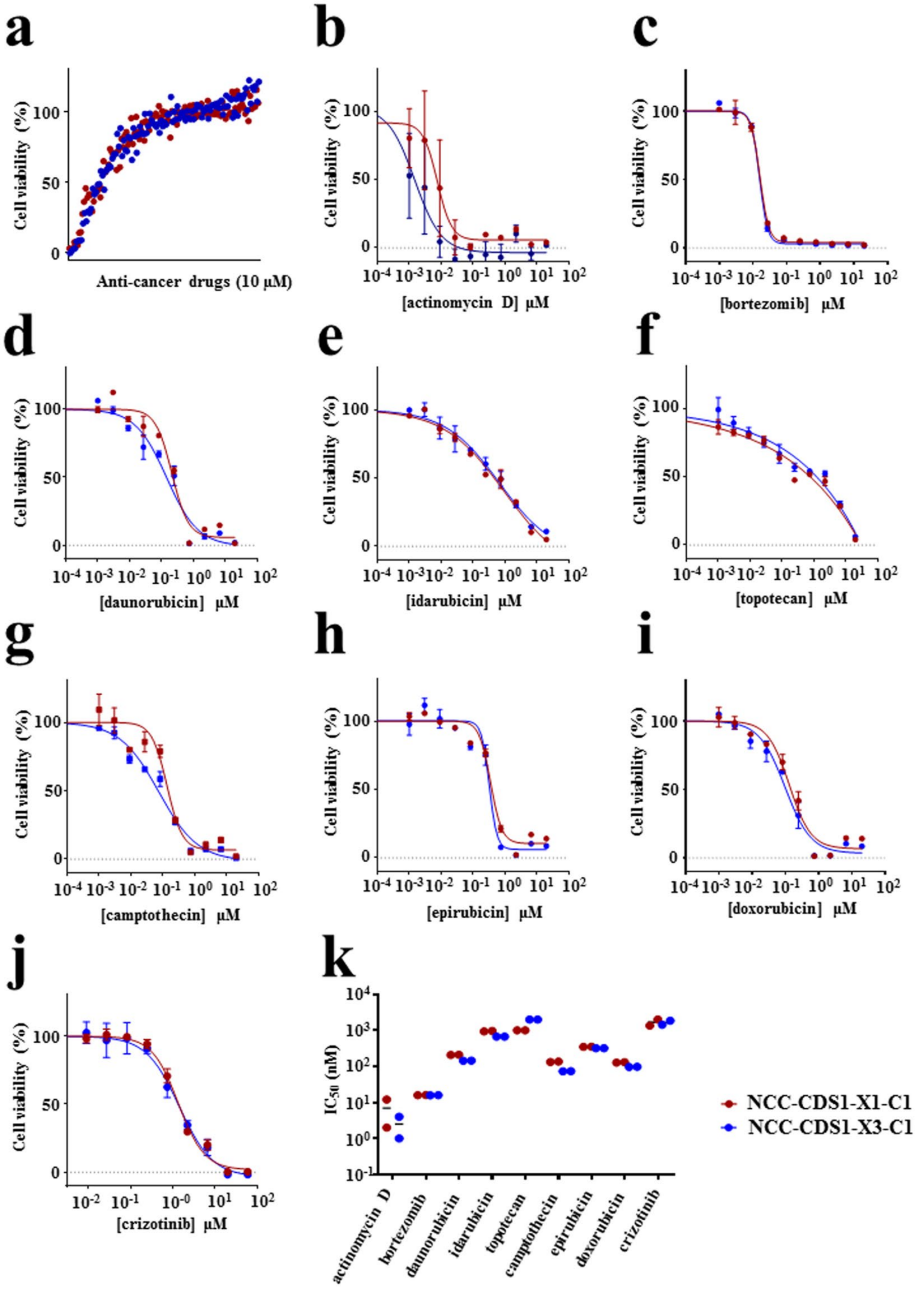
Anti-cancer drug screening in CDS cells. (**a**) NCC-CDS1-X1-C1 and X3-C1 cells were treated with 119 FDA approved anti-cancer compounds (10 μM) for 72 h. (**b–j**) Viability of the cells treated with anti-cancer drugs, including actinomycin D, bortezomib, daunorubicin, idarubicin, topotecan, camptothecin, epirubicin, doxorubicin, and crizotinib. (**k**) The IC_50_ value is shown for each anti-cancer drug.

The highlight of this study is the screening of anti-cancer drugs in the two cell lines. First, we screened 119 anti-cancer drugs at a fixed concentration (10 µM, Fig. 6a). When the threshold was set at 30% cell viability, nine drugs displayed remarkable growth-suppressive effects on the two cell lines, including actinomycin D, bortezomib, daunorubicin, idarubicin, topotecan, camptothecin, epirubicin, doxorubicin, and crizotinib (see Supplementary Dataset 6). We then determined the half maximal inhibitory concentration values (IC_50_) using four-parameter logistic curve fitting under threefold dilutions (Fig. 6b–j). No considerable differences were observed in the IC_50_ values across the nine anti-cancer drugs between the two cell lines (Fig. 6k). Doxorubicin was one of the most effective drugs in CDS cells. Notably, actinomycin D and doxorubicin are included in the standard therapeutic regimens for Ewing sarcoma, such as VACD (vincristine, actinomycin D, cyclophosphamide, and doxorubicin)^25^ and VDC-IE (vincristine, doxorubicin, cyclophosphamide, ifosfamide, and etoposide)^9^. The chemotherapy reagents used in VACD and VDC-IE treatments other than actinomycin D and doxorubicin did not show considerable growth-suppressive effects on CDS cells (see Supplementary Dataset 6). These observations strongly suggest the requirement of novel therapeutic strategies for CDS, which is different from Ewing sarcoma.

## Discussion

Patient-derived cancer models are an essential tool to establish novel therapeutic strategies. The present study established two cell lines, NCC-CDS1-X1-C1 and NCC-CDS1-X3-C1, derived from the xenograft NCC-CDS1-X1 and NCC-CDS1-X3 tumours, respectively. To our best knowledge, this is the first report on the establishment of patient-derived CDS cancer models. We characterised the established models, and examined the effects of existing anti-cancer drugs on the cells.

The histological appearances of two xenograft tumours were quite similar to those of the original tumour (Fig. 2). In contrast, the tumour proliferation was accelerated during the xenograft passage, while the third-passage xenograft presented a higher proliferative ability than the first passage one. A previous study reported that the human stromal components in the tumour tissues were replaced by those from mouse during the xenograft passage^26^. Such alterations may explain the different growth rates of the two xenograft models. In addition, some stromal components might be replaced by tumour cells. Probably reflecting such differences, the proteomic profiles of the two xenograft tumours were different. For instance, the focal adhesion pathway was enriched only in the first-generation xenograft and disappeared in the third passage, and the expression of such genes might be affected by the alterations of stromal components. The cell line established from the third-passage xenograft tumour, NCC-CDS1-X3-C1, exhibited a shorter population doubling time than that derived from the first xenograft passage, NCC-CDS1-X1-C1 (Fig. 3c and d). We assume that more aggressive and faster growing tumour cells were selected during the xenograft passaging. The selection of tumour cells in xenografts may also explant the different kinase activity profiles (Fig. 4). We did not find any other differences between the first- and third-passage xenografts, and between the derived cell lines.

The presence of *CIC-DUX4* in-frame fusion transcript was confirmed in primary tumour, xenograft tumours, and the two cell lines (Figs 1 and 3). *CIC* breakpoint of the fusion transcript was coincident with the insertion of a short fragment. These results were similar to that previously reported by Italiano *et al*.^6^, which identified a 14-nucleotide insertion in the antiparallel 4760–4782 region of *CIC* exon 20. The deduced amino acid sequence of the chimeric protein indicated that most of the CIC protein was preserved, while a large part of the N-terminal region of DUX4 was deleted, consistent with that observed by Kawamura-Saito *et al*.^4^. Moreover, *CIC-DUX4* sarcomas are characterised by consistent ETV1, ETV4, and ETV5 upregulation, and ETV4 immunohistochemistry has been identified as a highly sensitive marker for this tumour entity^27^. Accordingly, we confirmed the homogenous expression of ETV4 in both two cell lines.

The CIC HMG-box recognises and binds octameric T(G/C)AATG(A/G)A sites in target promoters and enhancers to repress transcription^4, 15, 28–31^. In *Drosophila*, repression by CIC appears to be tightly coupled to the RTK-dependent transcriptional control. CIC silences RTK genes, and when RTK signalling is activated, the CIC repression is relieved and gene expression is induced^13–16, 32–34^. Using the substrate peptides array of tyrosine kinases, we evaluated the activities of RTK and other tyrosine kinases. Although overall features of phosphorylated peptides were mostly similar among the samples examined, phosphorylation signals were highest in the primary and third passage xenograft tumours (Fig. 4a). As expected, kinases from first- and third-passage xenograft tumours and cell lines phosphorylated the Src family non-receptor tyrosine kinase substrate peptides (Fig. 4a). In addition, western blotting clearly indicated increased expression of active Src in third-generation xenografts as compared to that in the first-generation ones, whereas weak signals were detected in both cell lines (Fig. 4b). These observations suggest that Src kinase activity is up-regulated and modified during the process of CDS model establishment.

The spectrum of pathways enriched in the various tumour tissues and established cell lines suggest that protein expression profiles are continuously changing. Thus, the efficacy of anti-cancer drugs targeting pathways enhanced in cancer cell line models may be different from that observed *in vivo*, emphasizing the need to incorporate biological profiles influenced by the environmental differences in therapeutic drug development. The integration of multiomics data other than the proteome, such as genome and transcriptome data, will also be helpful to effectively utilize these established patient-derived cancer models.

Interestingly, except for doxorubicin and actinomycin D, the standard chemotherapy drugs to treat Ewing sarcoma showed no growth-suppressive effects on CDS cells. These observations further emphasise the need for novel therapeutic strategies for patients with CDS. Two other molecular target drugs exhibited growth-suppressive effects on the two cell lines. Bortezomib is a 26S proteasome inhibitor that binds the active site with high affinity and specificity, and currently used to treat multiple myeloma^35^. Bortezomib imparted growth inhibitory effects on Ewing sarcoma cell lines^36^, and combination treatment with the HSP90 inhibitor PU-H71 significantly reduced the growth of Ewing sarcoma xenografts^36^. In addition, crizotinib—a potent inhibitor for anaplastic lymphoma kinase (ALK), hepatocyte growth factor receptor (HGFR, c-MET), ROS1, and Recepteur d’Origine Nantais (RON)—was the first ALK-inhibitor and approved for the treatment of ALK-positive advanced non-small cell lung cancer^37, 38^. Moreover, a previous report demonstrated that crizotinib reduced cell viability in Ewing sarcoma^39^. Thus, our results suggest the possible re-purposing of these molecular inhibitors to treat patients with CDS. Most importantly, further investigation of the biological background underlying CDS cell sensitivity for these treatments may lead to the identification of novel predictive biomarkers, which will optimise therapeutic strategies for CDS.

Our PDX and cell lines may also be a useful resource to investigate the tumorigenesis of CDS. Previous reports demonstrate that differences in ERK signalling pathway may regulate kinase inhibitor resistance in CIC^40, 41^; thus, it is likely that the functional significance of *CIC-DUX4* in TKI sensitivity and resistance could be determined with our cell lines.

Our study had several limitations. First, we established our patient-derived CDS model from only one clinical case; thus, our findings should be validated in models generated from additional CDS cases. Second, we identified nine candidate anti-CDS drugs by an *in vitro* analysis using the two cell lines; however, these effects should also be verified in the xenograft models before application in clinical trials. The use of multiple cell lines may be a candidate approach to prioritize the candidate anti-cancer drugs. Thirdly, we performed mass spectrometry to elucidate proteomic alterations incurred during the establishment of patient-derived xenografts and cell lines. Although we found that several important biological pathways were drastically changed, this approach does not cover the entire proteome and more comprehensive data will be necessary to obtain conclusive results. Thus, the combined use of proteomic modalities with other omics techniques will further our understanding of mechanisms underlying the generation of patient-derived xenografts and cell lines, and serve as an informative resource for the future development of CDS models.

In conclusion, the present study describes the establishment of patient-derived CDS xenograft models and two cell lines that will be useful resources for further investigation on molecular carcinogenesis and the development of novel strategies to treat patients with CDS.

## Methods

### Patient’s background

A 29-year-old female was referred to the National Cancer Center Hospital with complaints of a soft tissue mass present in her sole. No metastasis was found at the initial assessment. Excisional biopsy was performed. On the basis of histological findings and FISH results, the patient was diagnosed with *CIC*-rearranged sarcoma and received surgical wide resection with postoperative chemotherapy using doxorubicin and ifosfamide. The patient achieved continuous disease-free survival for 1 year after completing chemotherapy. This study was conducted in accordance with the guidelines of the Ethics Committee of National Cancer Center and written informed consent was obtained from the patient. The experimental protocols involving human patients were approved by National Cancer Center and licensing committee by including a statement.

### Development of the CDS tumour xenograft

Several 2–3-mm pieces of CDS tissue were subcutaneously implanted with a 13-gauge transplant needle into the hind bilateral flanks of 6–12-week-old female severe immunodeficient NOD. Cg-*Prkdc* ^*scid*^ *Il2rg* ^*tm1Sug*^ */Jic* (also known as NOD/Shi-*scid IL-2Rγ* ^*null*^ or NOG) mice (Central Institute for Experimental Animals). When the tumours reached 500–1000 mm^3^, they were harvested and subsequently transplanted into another recipient mice. Tumour size was measured periodically using a digital calliper (SuperCaliper, Mitutoyo). Tumour volume was calculated as π/6 × length × width × thickness. After two passages, the tumours were cryopreserved using Cellbanker 1 plus (Takara Bio) in liquid nitrogen. All animal experiments were performed in accordance with the guidelines for Animal Experiments of the National Cancer Center and approved by the Institutional Committee for Ethics of Animal Experimentation.

### Establishment of a novel CDS cell line

Xenograft tumour tissue were cut into small pieces in RPMI-1640 medium (Sigma-Aldrich) supplemented with 10% fetal bovine serum (FBS) (Gibco), 100 U penicillin G, and 100 µg/mL streptomycin (Gibco). The small tissue pieces were treated with collagenase 2 (Worthington) at 37 °C for 5 min and then dissociated mechanically by passage through an 18-gauge (1.02 mm) needle. Cell suspensions were filtered with a 40-μm nylon mesh (BD Falcon) and seeded in a 10-cm culture plate at 37 °C in a humidified atmosphere of 5% CO_2_. Weakly adherent cells were maintained for >12 months in culture and passaged >50 times. Cells continuously expressed the *CIC*-*DUX4* transcript throughout establishment, as determined by RT-PCR. The absence of contaminated mycoplasma was examined with e-Myco Mycoplasma PCR Detection Kit (Intron biotechnology). Cell lines were authenticated with GenePrint 10 (Promega).

### FISH analysis

FISH analysis was performed on 4-µm-thick sections. A solid cell pellet was prepared from adherent tumour cells by iPGell (Geno Staff). Break-apart probes were used for *CIC* (custom-made probe; Chromosome Science Labo) genes as previously described^12^. FISH images were obtained using the Metafer Slide Scanning Platform (MetaSystems). The presence of split 5′ and 3′ signals or isolated 5′ signals in more than 20% of tumour cells was considered positive for *CIC* rearrangement.

### Genetic analysis

Genomic DNA and total RNA were extracted from excised tissue or cultured cells with the AllPrep DNA/RNA mini kit (Qiagen). Total RNA (1 μg) was used for the reverse transcription (RT) reaction with Superscript III reverse transcriptase (Invitrogen) according to the manufacturer’s instructions. The *CIC–DUX4* fusion transcript was amplified with *CIC* forward primer CICF4120 (5′-TGAGTTGCCTGAGTTTCG-3′) and *DUX4* reverse primer DUX4RTr2 (5′-TGAGGGGTGCTTCCAGCG-3′), using Q5 High-Fidelity DNA polymerase (New England Biolabs). For Sanger sequence analysis, the products were further amplified with forward (CIC2F; 5′-ATCATGCAGGCTGCCACT-3′) and reverse DUX4R2 (5′-ATGCCTTGCATCTGCCC-3′) primers for junction, or with reverse DUX4-R1 (5′-TCTAGGAGAGGTTGCGCCTG-3′) primer to determine whether the fusion gene was *DUX4* (4q) or *DUX4 L* (10q), respectively. The PCR products were purified with ExoSAP-IT (Affymetrix) and direct sequencing was performed using BigDye v3.1 Cycle Sequencing Kit (Applied Biosystems) on the Applied Biosystems 3130xL by Eurofin genomics (Japan). The sequence data were matched against the *CIC* (NM_015125.4) and *DUX4* (NM_001293798.2) sequences, using BLAST (NIH).

### Cell proliferation assay

Cells passaged over 30 times were seeded in triplicates in 96-well culture plates with RPMI-1640 medium containing 10% FBS and cultured at 37 °C under 5% CO_2_. Proliferation was measured by the Cell Counting Kit 8 (CCK-8, Dojindo Molecular Technologies). Spheroids were generated from cell suspension. The cells were subsequently prepared by filtration using a 40-μm filter after dissociation with dispase I (Godo Shusei), and 1 × 10^6^ cells were spread in 6-cm plate (ultra-low attachment, Thermo Fisher Scientific) in RPMI-1640 medium with 10% FBS.

### Histological analysis

Tumour tissue samples were fixed in 10% buffered formalin and embedded in paraffin. The 4-μm-thick sections were stained with haematoxylin and eosin. The cells were suspended, embedded in iPGell, fixed with 4% paraformaldehyde in prior ﻿to paraffin embedded, and then sections were subjected to heat-induced epitope retrieval. Antibodies against WT1 (6F-H2, 1:50, Dako) and ETV4 (clone 16, dilution 1:50, Santa Cruz Biotechnology) were used. Antigen-antibody reactions were visualized using the EnVision system (Dako).

### PamGene tyrosine kinase array

The kinase activity was measured by PamStation according to the instruction manual (Pamgene). Briefly, the protein lysates from the tumour tissue, xenograft tumour tissue, and cultured cells were prepared using M-Per mammalian protein extraction reagent with proteases and phosphatase inhibitors (Thermo Fisher Scientific). The lysates were kept at −80 °C until use. Protein concentration was determined using a Protein assay kit (BioRad). Tyrosine kinase PamChips (PamGene) were treated with 2% bovine serum albumin (fraction V, Thermo Fisher Scientific), and then 10 μg of protein was applied to the chip with standard kinase buffer, 100 μM ATP, and monoclonal FITC-conjugated anti-phosphotyrosine (clone Py20, Abcam) by pumping the solution through the porous microarray for 30 cycles of 30 s. The sample solution was pulsed back and forth through the porous material for 60 cycles. The fluorescence signals were analysed with BioNavigator software (PamGene). Fluorescence signals were normalised by the ‘vsn’ method, which allows missing value and, therefore, uses the ArcSinh instead of the logarithm for variance stabilisation. All analyses were performed with R software (version 3.3.0). The study was performed in triplicate. Kinases that phosphorylate substrate peptides were predicted by referring to Phospho.elm (http://phospho.elm.eu.org) and Uniprot (http://www.uniprot.org) databases.

### Western blotting

Cell lysates (20 μg) were separated on 12.5% SDS–PAGE gels, and transferred to Immuno-blot PVDF membranes (BioRad). After blocking with 5% skim milk in a TBS-Tween 0.1% solution, the membrane was reacted with anti-phospho-Src (Tyr416; dilution 1:1000) and anti-Src (36D10; dilution 1:1000) antibodies (all from Cell Signaling Technology) at room temperature for 1 h. Anti-actin (Abcam) was used as a loading control. Membranes were subsequently incubated with goat anti-mouse or anti-rabbit HRP-conjugated IgG (Jackson ImmunoResearch laboratories) for 1 h and bound HRP was detected using a Western lightning plus-ECL kit (Perkin Elmer).

### Screening of anti-cancer drugs, using CDS cell lines and cell sensitivity analysis

Cells (5000/well) were seeded in duplicate in a 384-well culture plate in RPMI-1640 medium containing 10% FBS and cultured overnight at 37 °C with 5% CO_2_. Ten micromolar of 119 FDA-approved anti-cancer drugs (Selleck Chemicals) were added to the cells, using the Bravo Automated liquid handling platform (Agilent technologies), and cell viability was assessed 72 h later with a CCK-8 kit.

To determine the cell sensitivity to the drugs, serially diluted anti-cancer drugs in RPMI-1640 medium containing 10% FBS were added to cells and cultured for 72 h. Cell viability was then assessed by CCK-8. IC_50_ values were calculated by drawing four-parameter curve fitting using GraphPad Prism (version 7, Graphpad Software). The study was performed in duplicate.

### Tryptic digestion of proteins and LC-Mass spectrometric protein expression profiling

Proteins were digested using a FASP protein digestion kit (Expedeon). Briefly, 25 μg of proteins were alkylated with iodoacetamide and then digested with trypsin in 50 mM NH_4_HCO_3_ at 37 °C overnight. The digested peptides were recovered with the NH_4_HCO_3_ and 0.5 M NaCl, and dried using a SpeedVac concentrator (Thermo Fisher Scientific). The peptides were dissolved and separated on an analytical column (C18, 3 μm, 100 Å, 150 × 0.075 mm, AMR) by nano-flow HPLC system (AMR) in a linear gradient with 0.1% formic acid (A) and 0.1% formic acid in 90% acetonitrile (B) of 5–45% B in 140 min at flow rate of 250 nL/min. The eluted peptides were ionized in the nano-spray ion source, and detected by a LTQ Orbitrap XL mass spectrometer (Thermo Fisher Scientific). Mass and tandem mass spectra data were used for peptide identification with the SWISS-PROT database (*Homo sapiens*, 20,205 sequences in the Swiss Prot_2015_09.fasta file) and Mascot v.2.5.1 software (Matrix Science). The search parameters were as follows: tolerance of one missed trypsin cleavage; variable modifications on the methionine (oxidation, +16 Da), and serine, threonine, and tyrosine (phosphorylation, +80 Da); fixed modifications on cysteine (carbamidomethly, +57 Da); maximum precursor ion mass tolerance of ±10 ppm; and a fragment ion mass tolerance of ±0.8 Da.

### Functional annotation of proteomic data

KEGG pathway annotations for each protein group identified from the MS data were inferred using Database for Annotation, Visualization and Integration Discovery (DAVID) software (http://david.abcc.ncifcrf.gov/). The KEGG database was used for the classification of correlating gene sets into their respective pathways. Significance is indicated by the p-value for each category and the process groups were considered significant with p < 0.05. KEGG analysis results were plotted using the R package “treemap”^42^.

## Electronic supplementary material

Supplementary information

Dataset 1

Dataset 2

Dataset 3

Dataset 4

Dataset 5

Dataset 6
