# Supplementary material for: Generation of novel patient-derived CIC-DUX4 sarcoma xenografts and cell lines
Source: Sci Rep. 2017 Jul 5;7:4712. doi: 10.1038/s41598-017-04967-0 (PMC5498486; doi:10.1038/s41598-017-04967-0)
Supplement: Supplementary file 1 — Supplementary information [file 41598_2017_4967_MOESM1_ESM.pdf]

**Supplementary Information**

# Generation of novel patient-derived *CIC-DUX4* sarcoma xenografts and cell lines

Rieko Oyama[1], Mami Takahashi[2], Akihiko Yoshida[3], Marimu Sakumoto[1], Yoko Takai[1], Fusako Kito[1], Kumiko Shiozawa[4], Zhiwei Qiao[4], Yasuhito Arai[5], Tatsuhiro Shibata[5], Yoshihiro Araki[6], Makoto Endo[6], Akira Kawai[6], and Tadashi Kondo[1, 4*]

[1]Department of Innovative Seeds Evaluation, National Cancer Center Research Institute, 5-1-1 Tsukiji, Chuo-ku, Tokyo 104-0045, Japan

[2]Central Animal Division, National Cancer Center Research Institute, 5-1-1 Tsukiji, Chuo-ku, Tokyo 104-0045, Japan–

[3]Pathology and Clinical Laboratory Division, National Cancer Center Hospital, 5-1-1 Tsukiji, Chuo-ku, Tokyo 104-0045, Japan

[4]Division of Rare Cancer Research, National Cancer Center Research Institute, 5-1-1 Tsukiji, Chuo-ku, Tokyo 104-0045, Japan

[5]Division of Cancer Genomics, National Cancer Center Research Institute, 5-1-1 Tsukiji, Chuo-ku, Tokyo 104-0045, Japan

[6]Division of Musculoskeletal Oncology, National Cancer Center Hospital, 5-1-1 Tsukiji, Chuo-ku, Tokyo 104-0045, Japan

**Table S1.** STR analysis

| Microsatellite (Choromosome) | NCC-CDS1-X1-C1 (P14) | NCC-CDS1-X2-C1 (P35) | NCC-CDS1-X1 (PDX) | NCC-CDS1-X2 (PDX) | Tumor Tissue |
|---|---|---|---|---|---|
| Amelogenin (X Y) | X,X | X,X | X,X | X,X | X,X |
| TH01 (3) | 7,9 | 7,9 | 7,9 | 7,9 | 7,9 |
| D21S11 (21) | 30,30 | 30,30 | 30,30 | 30,30 | 30,30 |
| D5S818 (5) | 10,12 | 10,12 | 10,12 | 10,12 | 10,12 |
| D13S317 (13) | 11,12 | 11,12 | 11,12 | 11,12 | 11,12 |
| D7S820 (7) | 12,13 | 12,13 | 12,13 | 12,13 | 12,13 |
| D16S539 (16) | 9,13 | 9,13 | 9,13 | 9,13 | 9,13 |
| CSF1PO (5) | 11,13 | 11,13 | 11,13 | 11,13 | 11,13 |
| vWA (12) | 14,16 | 14,16 | 14,16 | 14,16 | 14,16 |
| TPOX (2) | 8,8 | 8,8 | 5,8 | 8,8 | 8,8 |

Cells were compared to a normal and tumor tissue from the patient.

P indicates a passage number of a cell line.

**Table S2.** KEGG pathways enriched in the proteome of different samples.

| KEGG Enrichment Rank | Pathway | Primary tumour | NCC-CDS1-X1 | NCC-CDS1-X3 | X1-C1 | X3-C1 | Observations |
|---|---|---|---|---|---|---|---|
| 1 | Proteasome | 1 | 2 | 2 | 3 | 3 | Consistently enriched in all samples |
| 2 | Spliceosome | 2 | 3 | 3 | 2 | 2 | |
| 3 | Ribosome | 3 | 1 | 1 | 1 | 1 | |
| 4 | Pathogenic Escherichia coli infection | 4 | 4 | 5 | 5 | 7 | |
| 5 | Glycolysis / Gluconeogenesis | 5 | 6 | 4 | 8 | 8 | |
| 8 | Pyruvate metabolism | 8 | 8 | 9 | 9 | 9 | |
| 9 | Huntington's disease | 9 | 15 | 13 | 6 | 6 | |
| 12 | Pentose phosphate pathway | 12 | 7 | 6 | 15 | 21 | |
| 14 | Citrate cycle (TCA cycle) | 14 | 5 | 7 | 4 | 4 | |
| 19 | Glyoxylate and dicarboxylate metabolism | 19 | 9 | 11 | 22 | 12 | |
| 17 | Aminoacyl-tRNA biosynthesis | 17 | | 16 | 7 | 5 | Consistintly enriched in all but NCC-CDS1-X1 samples |
| 18 | Parkinson's disease | 18 | | 15 | 10 | 10 | |
| 24 | Alzheimer's disease | 24 | | 20 | 11 | 13 | |
| 6 | Focal adhesion | 6 | 10 | 21 | | | Enriched in all but xenograft tissues and cell lines |
| 25 | Regulation of actin cytoskeleton | 25 | 12 | | | | |
| 23 | Tight junction | 23 | | 14 | | | |
| 15 | Fatty acid elongation in mitochondria | 15 | | | 16 | 16 | Enriched in xenograft tissues and cell lines |
| 16 | Fatty acid metabolism | 16 | | | 20 | 11 | |
| 20 | Methane metabolism | 20 | | | | 20 | |
| 29 | Propanoate metabolism | | 11 | | 17 | | Irregularly enriched in primary tumours and cell lines |
| 35 | DNA replication | | | 17 | 18 | | |
| 33 | Cysteine and methionine metabolism | | | 8 | | 19 | |
| 7 | Complement and coagulation cascades | 7 | | | | | Enriched only in primary tumor tissue |
| 10 | Systemic lupus erythematosus | 10 | | | | | |
| 11 | Prion diseases | 11 | | | | | |
| 13 | Antigen processing and presentation | 13 | | | | | |
| 21 | Valine, leucine and isoleucine degradation | 21 | | | | | |
| 22 | Viral myocarditis | 22 | | | | | |
| 27 | Gap junction | | 14 | 12 | | | Enriched only in xenograft tissues |
| 30 | Fructose and mannose metabolism | | 13 | 10 | | | |
| 31 | Cell cycle | | 16 | 18 | | | |
| 32 | Leukocyte transendothelial migration | | 17 | 25 | | | |
| 36 | One carbon pool by folate | | | 19 | | | |
| 45 | Lysine degradation | | | | 13 | 14 | Enriched only in cell lines, either consistently or irregularly |
| 46 | Valine, leucine and isoleucine biosynthesis | | | | 14 | 24 | |
| 48 | Arginine and proline metabolism | | | | 21 | 22 | |
| 28 | Fructose and mannose metabolism | | | | 19 | | |
| 41 | Oocyte meiosis | | | | 23 | | |
| 44 | Oxidative phosphorylation | | | | 12 | | |
| 47 | Butanoate metabolism | | | | 18 | | |
| 38 | Cardiac muscle contraction | | | | | 23 | |
| 39 | Protein export | | | | | 17 | |
| 51 | Oxidative phosphorylation | | | | | 15 | |
| 52 | Selenoamino acid metabolism | | | | | 25 | |

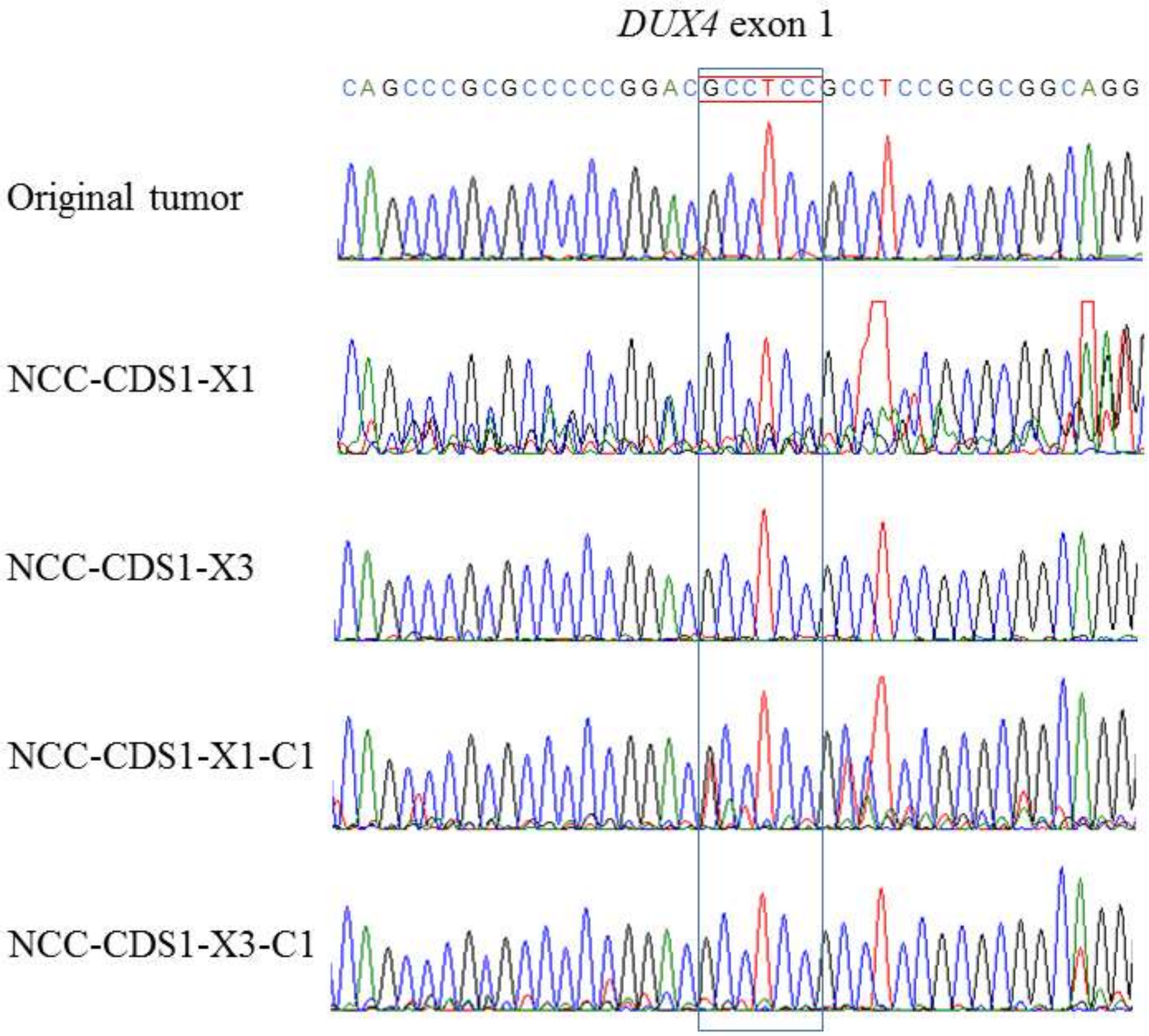


**Figure S1.** Sanger sequencing showed that the nucleotide sequence 5'-CGCCTC-3' was detected within *DUX4* exon 1, but not *DUX4L*, indicating that *CIC* was fused to *DUX4* (4q35). The sample names are shown on the left side of the panels.

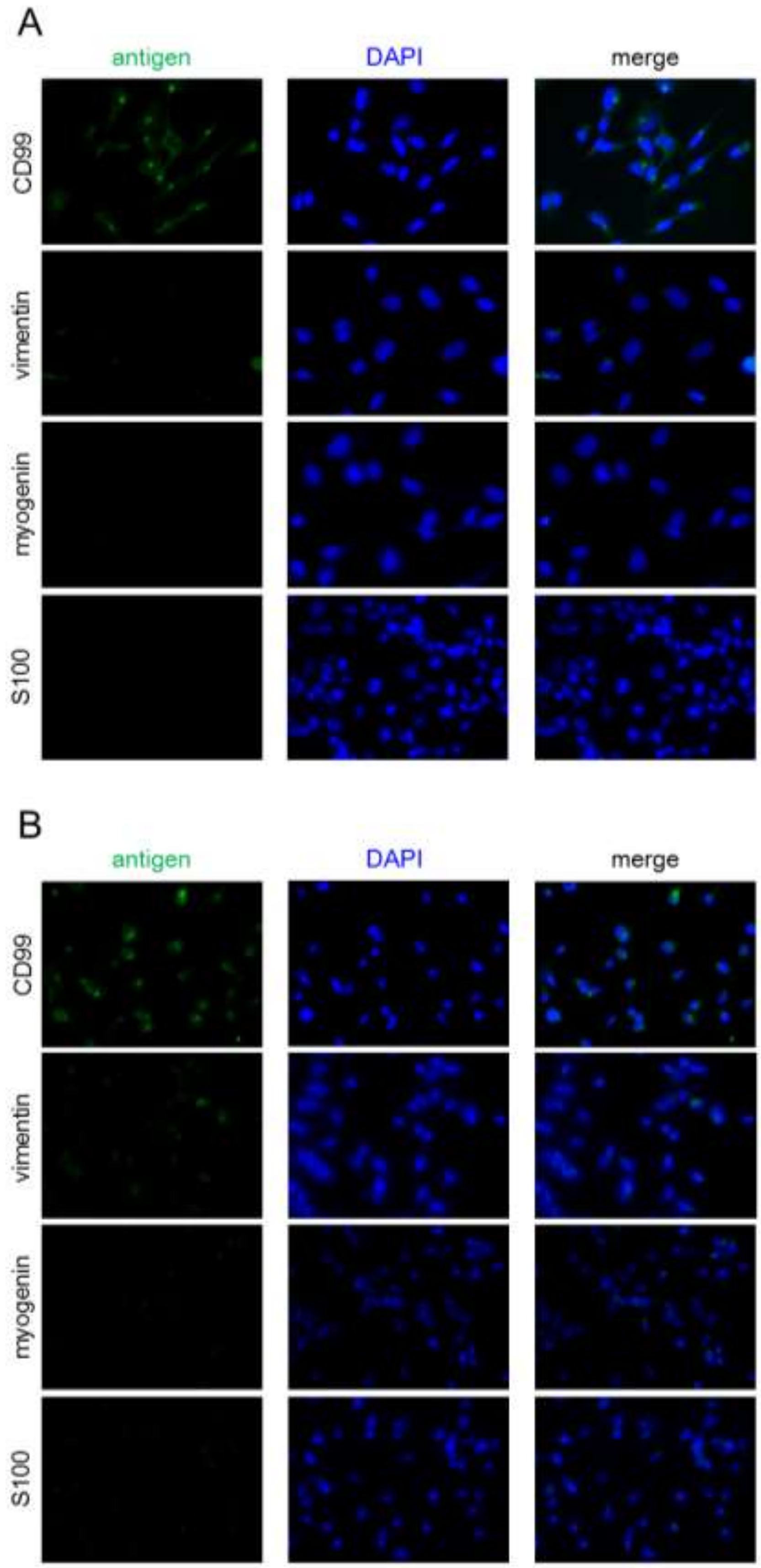


**Figure S2.** Immunofluorescence study for CD99, vimentin, myogenin, and S100. (**A**) NCC-CDS1-X1-C1, (**B**) NCC-CDS1-X3-C1.

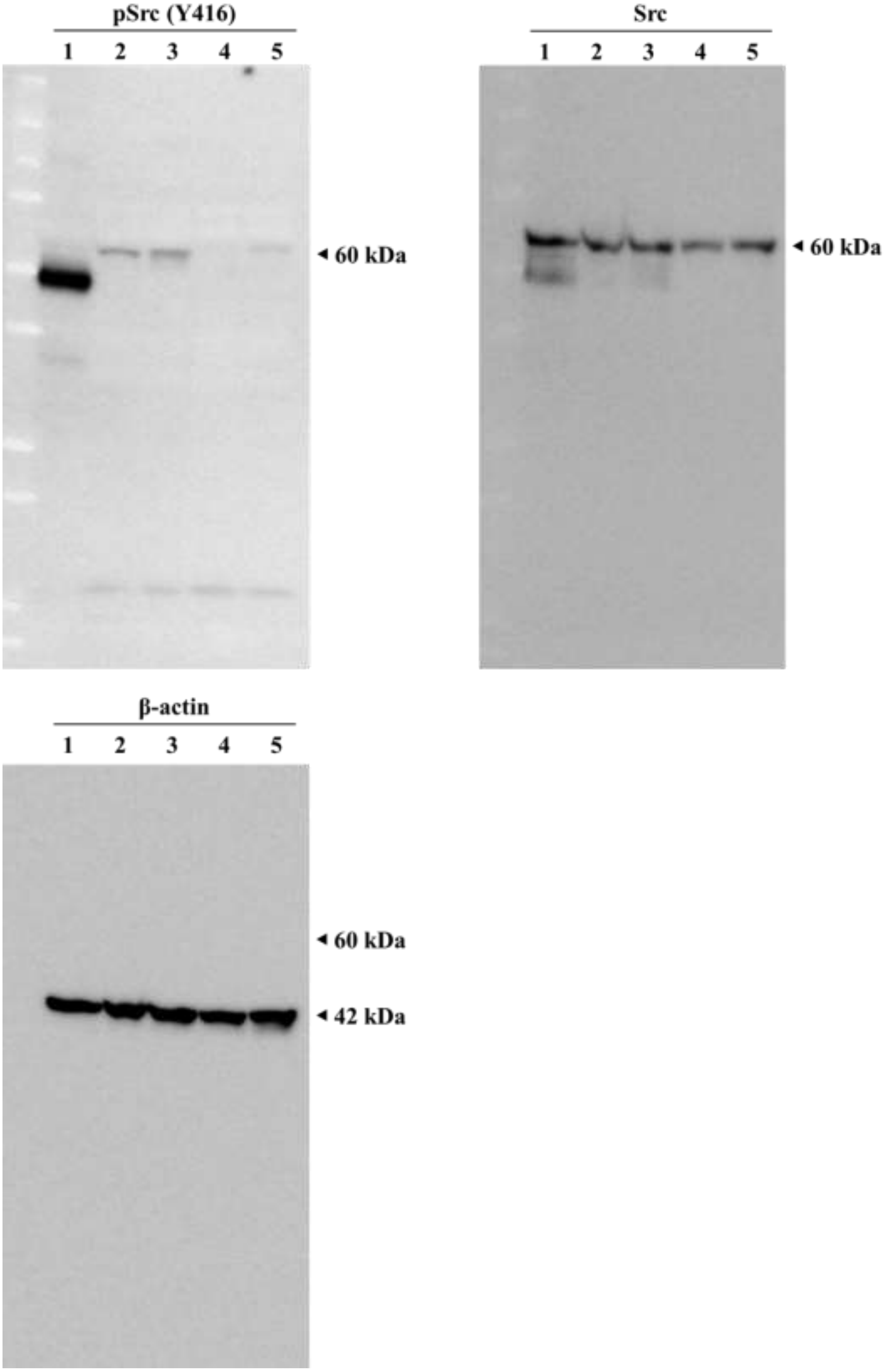


**Figure S3.** Phosphorylated Src expressions were analysed by Western blotting. Arrowheads indicate the SuppleTable S2, position of Src with a molecular weight of 60 kDa. Lanes 1: tumour tissue, 2: NCC-CDS1-X1 tumour tissue, 3: NCC-CDS1-X3 tumour tissue, 4: NCC-CDS1-X1-C1 cells, 5: NCC-CDS1-X3-C1 cells.
